# Sevoflurane inhibits cholangiocarcinoma via Wnt/β-catenin signaling pathway

**DOI:** 10.1186/s12876-023-02911-3

**Published:** 2023-08-11

**Authors:** Hui Cheng, Qinfang Li

**Affiliations:** 1 Department of Anesthesiology, People’s Hospital of Dongxihu District, Wuhan, 430040 China; 2 People’s Hospital of Dongxihu District, No. 81 Huanshan Road, Wujiashan, Dongxihu District, Wuhan, 430040 China

**Keywords:** Sevoflurane, Cholangiocarcinoma, Wnt/β-catenin pathway

## Abstract

**Background:**

Cholangiocarcinoma (CCA) is a refractory malignancy derived from bile duct epithelial cells. This study aimed to explore the role and molecular mechanisms of action of sevoflurane in CCA.

**Methods:**

CCK-8 assay was used to assess the proliferation of cholangiocarcinoma cells, and flow cytometry was used to detect cholangiocarcinoma cell apoptosis. The effects of sevoflurane on TFK1 and QBC939 cell migration and invasion were investigated using a Transwell assay. Western blotting and RT-qPCR were used to assess the expression of apoptosis-related proteins and genes, and gene expression of the Wnt/β-catenin signaling pathway.

**Results:**

Our study found that sevoflurane inhibited cholangiocarcinoma cell proliferation in a dose-dependent manner. In addition, sevoflurane induced cholangiocarcinoma cell apoptosis, inhibited cholangiocarcinoma cell migration and invasion, as well as the Wnt/β-catenin signaling pathway evidenced by decreased Wnt3a, β-catenin, c-Myc, and Cyclin D1 protein and mRNA expression, reduced p-GSK3β protein expression and p-GSK3β/GSK3β ratio. Further mechanistic studies revealed that Wnt/β-catenin pathway inducer SKL2001 reversed the inhibitory effect of sevoflurane on cholangiocarcinoma cells.

**Conclusions:**

Sevoflurane induces apoptosis and inhibits the growth, migration, and invasion of cholangiocarcinoma cells by inhibiting the Wnt/β-catenin signaling pathway. This study not only revealed the role of sevoflurane in the development of CCA but also elucidated new therapeutic agents for CCA.

**Supplementary Information:**

The online version contains supplementary material available at 10.1186/s12876-023-02911-3.

## Background

Cholangiocarcinoma (CCA) is a malignant tumor derived from the epithelial cells of the bile ducts [1, 2]. The incidence of CCA is higher in China than in Europe and the United States, with approximately 6 per 100,000 people suffering from CCA [1]. The pathogenesis of CCA remains unclear, but some reports suggest that CCA may be related to diseases such as bile duct stones and primary sclerosing cholangitis [3]. However, CCA has a high rate of metastasis and often metastasizes to nearby lymph nodes, resulting in high mortality rates [2, 4]. Currently, the only clinical treatment is surgical removal of the tumor [5]. However, the prognosis for surgical treatment of CCA is poor, with a survival rate of 20–40% in patients who undergo surgery [6]. Currently, there is no effective treatment for CCA, and it is necessary to explore new therapeutic drugs.

Sevoflurane is a widely used inhalation anesthetic drug in clinical practice that has good cerebral and myocardial protective effects [7, 8]. Studies have shown that sevoflurane inhibits cell proliferation, induces apoptosis, and inhibits tumorigenesis [9]. For example, recent studies have reported that sevoflurane regulates breast cancer development by activating the microRNA-203 signaling pathway to inhibit the proliferation of breast cancer cells [10]. In addition, sevoflurane regulates the development of gliomas as well as breast, lung, and colon cancers by inhibiting cell migration and invasion [10–13]. Studies have found Sevoflurane promotes the proliferation of colon cancer cells and inhibits the growth and migration of lung cancer cells [12, 14]. However, whether sevoflurane is involved in the development of CCA and its underlying mechanisms have not been fully elucidated.

Previous studies have shown that abnormal Wnt/β-catenin signaling is associated with human diseases, including tumors, osteoporosis, and degenerative disorders [15, 16]. Especially in cancer research, the Wnt/β-catenin signaling pathway has been implicated in a variety of cancer processes, and has been shown to be a therapeutic target for anti-tumor therapy [17, 18]. LINC01133 inhibits gastric cancer progression by modulating the-catenin pathway [19]. Recent studies have found that Wnt/β-catenin signaling is associated with the induction and progression of CCA and is a new potential pharmacological target for CCA [20]. Overexpression of RNF43 can attenuate the Wnt/β-catenin signaling pathway to inhibit the progression of CCA [21]. Song et al. found that mucin 1 promotes tumor progression by activating Wnt/β-catenin signaling pathway in CCA [22]. In addition, studies have found that sevoflurane inhibits the proliferation of neural progenitor cells in mice via Wnt/β-catenin signaling pathway [23, 24]. This suggests that sevoflurane may play a role in the CCA through Wnt/β-catenin signaling pathway.

In this study, we revealed the regulatory effects of sevoflurane on CCA and elucidated its effects on CCA proliferation and apoptosis. This study suggests that the Wnt/β-catenin signaling pathway may be a potential target for the prevention and treatment of CCA.

## Methods

### Cell culture and drug treatment

The cholangiocarcinoma cell lines TFK1 and QBC939 were purchased from the American Type Culture Collection (ATCC). To study the effects of sevoflurane on cholangiocarcinoma, we treated TFK1 and QBC939 cells with 1.7%, 3.4%, and 5.1% sevoflurane [25] for 2, 4, and 6 h. Cells were cultured in Dulbecco’s modified DMEM medium (Basal Media) containing 10% fetal bovine serum (FBS; Biological Industries) for 24 h. Treatment of TFK1 and QBC939 cells with 3.4% sevoflurane for 6 h was selected for subsequent experiments.

To investigate the role of Wnt/β-catenin signaling pathway in the effects of Sevoflurane on cholangiocarcinoma, we treated 3.4% Sevoflurane-treated TFK1 and QBC939 cells with 40 µM SKL2001 (Wnt/β-catenin inducer). The groups were as follows: Control; Sevoflurane; Sevoflurane + SKL2001.

### CCK-8 assays for cell proliferation

Cell proliferation was detected using a CCK-8 kit (Beyotime), according to the manufacturer’s instructions. Briefly, 2000 cells/well were seeded in a 96-well plate. After 24 h, 10 µl CCK-8 solution was added to each well. This was followed by incubation for 1 h in a 37 °C cell incubator. Absorbance was measured at 490 nm using a spectrophotometer.

### Flow cytometry detects apoptosis

Apoptosis was identified by flow cytometry using an Annexin V-FITC/PI Apoptosis Detection Kit (Beyotime). Briefly, drug-treated TFK1 and QBC939 cells were collected, and binding buffer containing 5 µL Annexin V and 10 µL PI was added. Data were collected using flow cytometry (BD Biosciences) after incubation in the dark for 10–20 min and analyzed using FlowJo software.

### Western blotting

Total protein was obtained from TFK1 and QBC939 cells using RIPA lysis buffer containing protein inhibitors (Beyotime), and total protein was assayed using a BCA kit (Beyotime). Subsequently, 20 µg of each sample was separated by SDS-PAGE and transferred onto polyvinylidene difluoride (PVDF) membranes (Millipore). The membranes were blocked with 5% bovine serum protein (BSA). After 1 h, membranes were incubated overnight at 4 °C with primary antibodies against Bax (#2772, CST), Bcl-2 (ab196495, Abcam), Wnt3a (26744-1-AP, Wuhan Sanying Biotechnology), β-catenin (#8480, CST), p-GSK3β (#5558, CST), GSK3β (#12,456, CST), c-Myc (ab32072, Abcam), Cyclin D1 (ab134175, Abcam), and GAPDH (ab181602, Abcam). The next day, membranes were washed five times with TBST and incubated with horseradish peroxidase-labeled secondary antibodies (AS1107, ASPEN). After 2 h, the bands were visualized using an ECL luminescent solution (Beyotime). However, during the western blot experiments, the corresponding membrane was firstly cut out according to the molecular weight of the target protein prior to hybridisation with antibody, and then incubated with the primary antibody. Thus, the original image was not a full length membrane.

### RNA extraction and real-time quantitative PCR (RT-qPCR)

Total RNA was extracted from TFK1 and QBC939 cells with an RNA-easy isolation reagent (Vazyme). RNA was reverse-transcribed to cDNA using the SuperScript™ III Reverse Transcription Kit (Thermo Fisher Scientific) according to the manufacturer’s instructions. This was followed by RT-qPCR using the AceQ qPCR SYBR Green Master Mix (Vazyme). The primers were synthesized by Sangon Biotech (Shanghai, China) with the following sequences: Bax forward 5’-GCTGAGCGAGTCTCTCAAG-3’ and reverse 5’-GTCCAATGTCCAGCCCATG-3’; Bcl-2 forward 5’-GGTGAACTGGGGGAGGATT G-3’ and reverse 5’-GGCAGGCATGTTGACTTCAC-3’; Wnt3a forward 5’-ATGGGCGGGAGG GGACA-3’ and reverse, 5’CGCCCATTGGATCCTTAAG3’; β-catenin forward 5’-CGTTTCGCCTTC ATGGACTA-3’ and reverse, 5’-GCCGCTGGGTCCTGATGTCCTGAT-3’; Cyclin D1 forward 5’-GCTGCGAAGTGGAAACCATC-3’ and reverse 5’-CCTCCTTCTGCACACATTTGAA-3’; c-Myc forward 5’-GCCTCAGAGTGCATCGAC-3’ and reverse 5’-TCCACAGAAACAACATCG-3’; GAPDH forward, 5’-GGAAGGTGAAGGTCGGAGTCA-3’ and reverse, 5’-GTCATTGATGGCAACAATCCACT-3’. The relative mRNA expression is calculated as 2^ΔΔCt^.

### Transwell assays to determine cell migration and invasion

Cell migration and invasion were assessed using Transwell assays [26]. Briefly, 1 × 10^5^ cells with serum-free culture medium were added to the upper layer of Transwell chambers with 8 μm pore size, while medium containing 10% serum was added to the lower layer of Transwell chambers. After 24 h, cells that penetrated the lower layer were fixed with 4% paraformaldehyde and stained with crystal violet. Finally, the migration and invasion of cells were counted using an inverted microscope (LEICA) under ×200 magnification.

### Statistical analysis

All data were analyzed using the GraphPad Prism 7 software. All data were obtained from at least three independent experiments and expressed as mean ± SEM. An unpaired Student’s t-test or one-way analysis of variance (ANOVA) was used to analyze differences between groups, and P < 0.05 indicated statistical significance.

## Results

### Sevoflurane inhibits proliferation and promotes apoptosis in cholangiocarcinoma cells

**Fig. 1:**
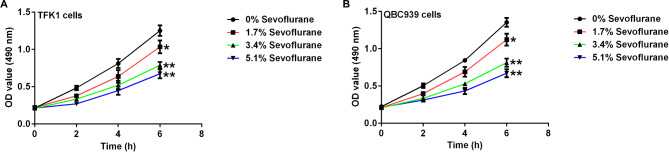
Effect of Sevoflurane on proliferation of CCA cells. **A-B**. CCK-8 assays were used to evaluate the cell proliferation of TFK1 **(A)** and QBC939 **(B)** cells. *, **p < 0.05, 0.001 vs. control group

To investigate the effect of sevoflurane on the proliferation of cholangiocarcinoma cells, we used the CCK-8 assay to detect changes in the proliferation and viability of TFK1 and QBC939 cells treated with different concentrations of sevoflurane (1.7%, 3.4%, and 5.1%). Compared with the control group, sevoflurane inhibited the viability of TFK1 and QBC939 cells, and the inhibitory effect of sevoflurane on the viability of TFK1 and QBC939 cells increased with increasing dose and treatment time (Fig. 1A and B). Based on these results, we treated TFK1 and QBC939 cells with 3.4% sevoflurane for 6 h for subsequent experiments.

**Fig. 2:**
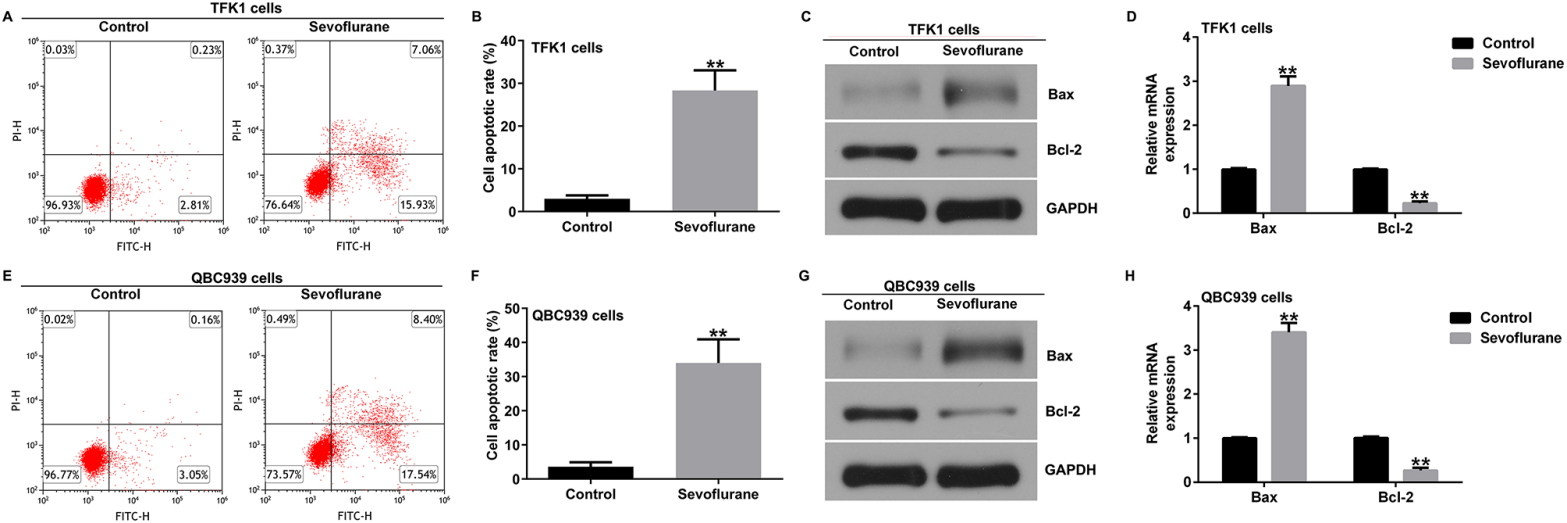
Effect of Sevoflurane on apoptosis of CCA cells. **A-B**. Flow cytometry were used to evaluate the apoptosis of TFK1 cells; **C** and **D**. Analysis of Bax and Bcl2 protein and mRNA expression in TFK1 cells by western blotting and RT-qPCR; **E** and **F**. Flow cytometry were used to evaluate the apoptosis of QBC939 cells; **G** and **H**. Analysis of Bax and Bcl2 protein and mRNA expression in TFK1 cells by western blotting and RT-qPCR. **p < 0.01 vs. control group

Flow cytometry indicated that sevoflurane significantly induced apoptosis in both TFK1 (Fig. 2A and B) and QBC939 cells (Fig. 2E and F). Further experiments showed that sevoflurane increased the protein and mRNA levels of Bax and decreased the protein and mRNA levels of Bcl-2 in TFK1 cells (Fig. 2 C and D). In QBC939 cells, Sevoflurane had the same effect on the expression levels of Bax and Bcl-2 (Fig. 2G and H). These results suggest that sevoflurane inhibits the proliferation of cholangiocarcinoma cells in a dose- and time-dependent manner and promotes apoptosis in cholangiocarcinoma cells.

### Sevoflurane inhibits migration and invasion of cholangiocarcinoma cells

**Fig. 3:**
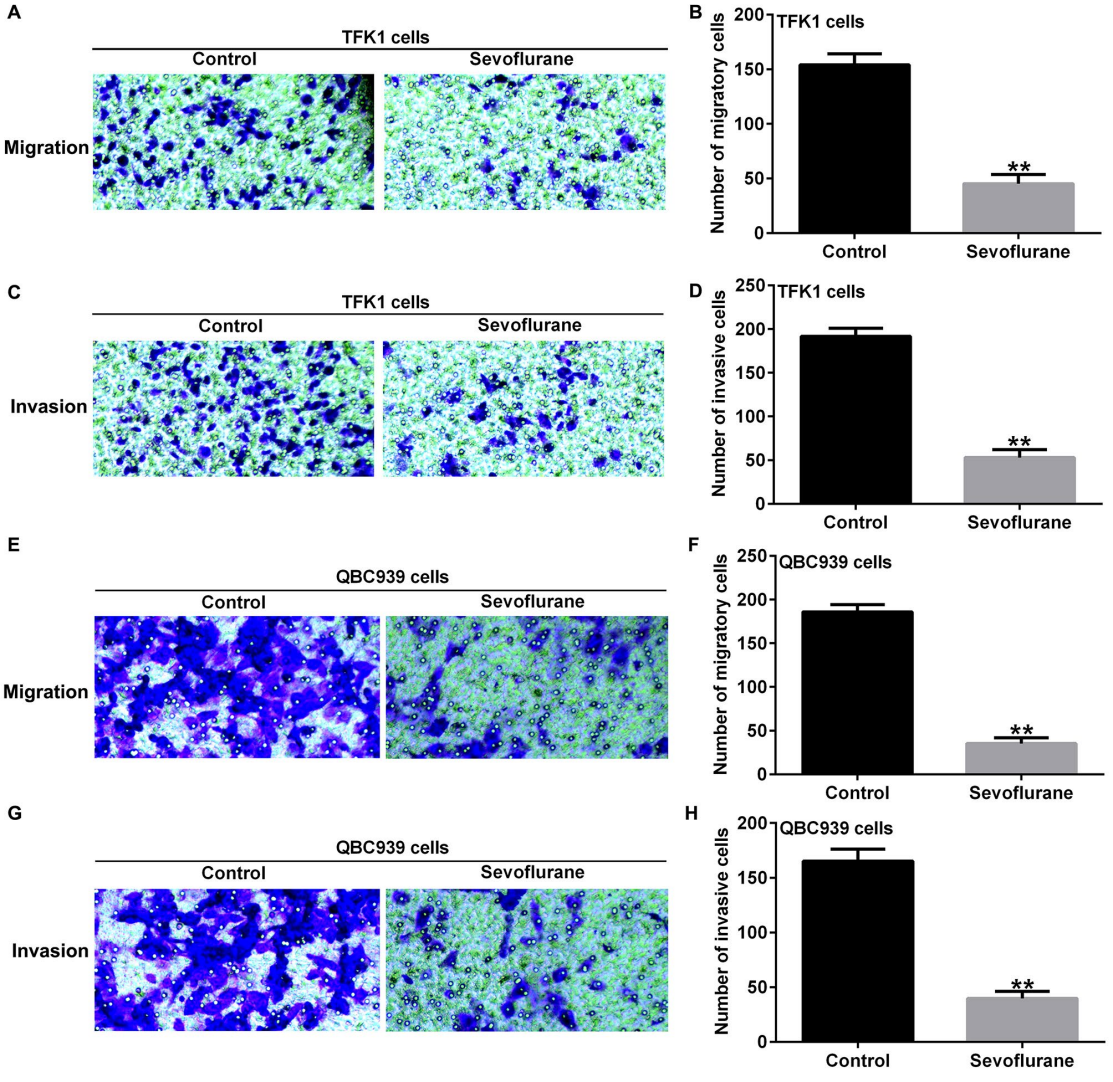
Effects of Sevoflurane on migration and invasion of CCA cells. **A-D**. Transwell assays were used to evaluate migration **(A-B)** and invasion **(C-D)** of TFK1 cells; E-H. Transwell assays were used to evaluate migration **(E-F)** and invasion **(G-H)** of QBC939 cells. Magnification: ×200. **p < 0.01 vs. control group

Subsequently, we treated TFK1 and QBC939 cells with 3.4% sevoflurane for 6 h and examined the effects of sevoflurane on the migration and invasion of cholangiocarcinoma cells using Transwell assays. The results indicated that 3.4% sevoflurane inhibited the migration (Fig. 3A and B) and invasion (Fig. 3 C and D) of TFK1 cells. Similarly, 3.4% sevoflurane significantly inhibited the migration (Fig. 3E and F) and invasiveness (Fig. 3G and H) of QBC939 cells. These results suggest that sevoflurane inhibits the migration and invasion of cholangiocarcinoma cells.

### Sevoflurane inhibits Wnt/β-catenin signaling pathway in cholangiocarcinoma cells

**Fig. 4:**
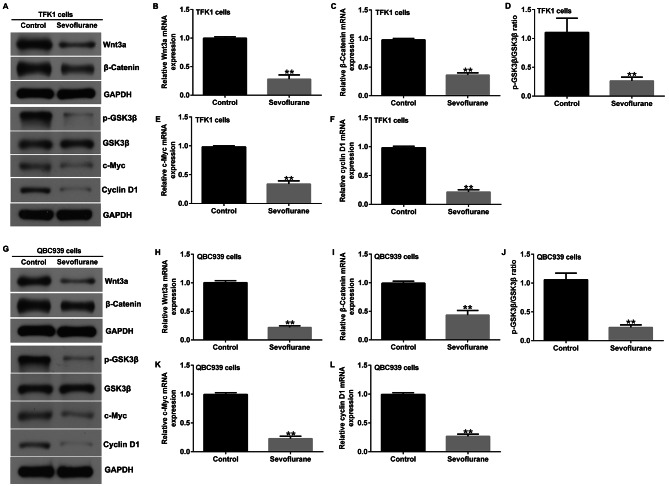
Sevoflurane inhibits Wnt/β-catenin Signaling in CCA cells. **A-F**. Western blotting and RT-qPCR detected the protein and mRNA expression of Wnt3a, β-catenin, p-GSK3β, GSK3β, c-Myc, and Cyclin D1 in TFK1 cells; **G-L**. Western blotting and RT-qPCR detected the protein and mRNA expression of Wnt3a, β-catenin, p-GSK3β, GSK3β, c-Myc, and Cyclin D1 in QBC939 cells. **p < 0.01 vs. control group

Recent studies have shown that-catenin signaling is an emerging potential target for CCA [20, 27]. Therefore, in this study, we analyzed the expression of catenin signaling-related genes in TFK1 and QBC939 cells using western blotting and RT-qPCR. Compared to the control group, the protein and mRNA levels of Wnt3a, β-catenin, c-Myc, and Cyclin D1, p-GSK3β protein expression, and p-GSK3β/GSK3β ratio in TFK1 cells were significantly reduced by 3.4% sevoflurane treatment for 6 h (Fig. 4A-F). Similarly, Sevoflurane inhibited the expression of Wnt3a, β-catenin, c-Myc, and Cyclin D1, p-GSK3β protein expression, and p-GSK3β/GSK3β ratio in QBC939 cells (Fig. 4G-L). These results suggest that sevoflurane inhibits Wnt/β-catenin signaling pathway in cholangiocarcinoma.

### Activation of Wnt/β-catenin signaling reverses the inhibitory effect of sevoflurane on cholangiocarcinoma cells

**Fig. 5:**
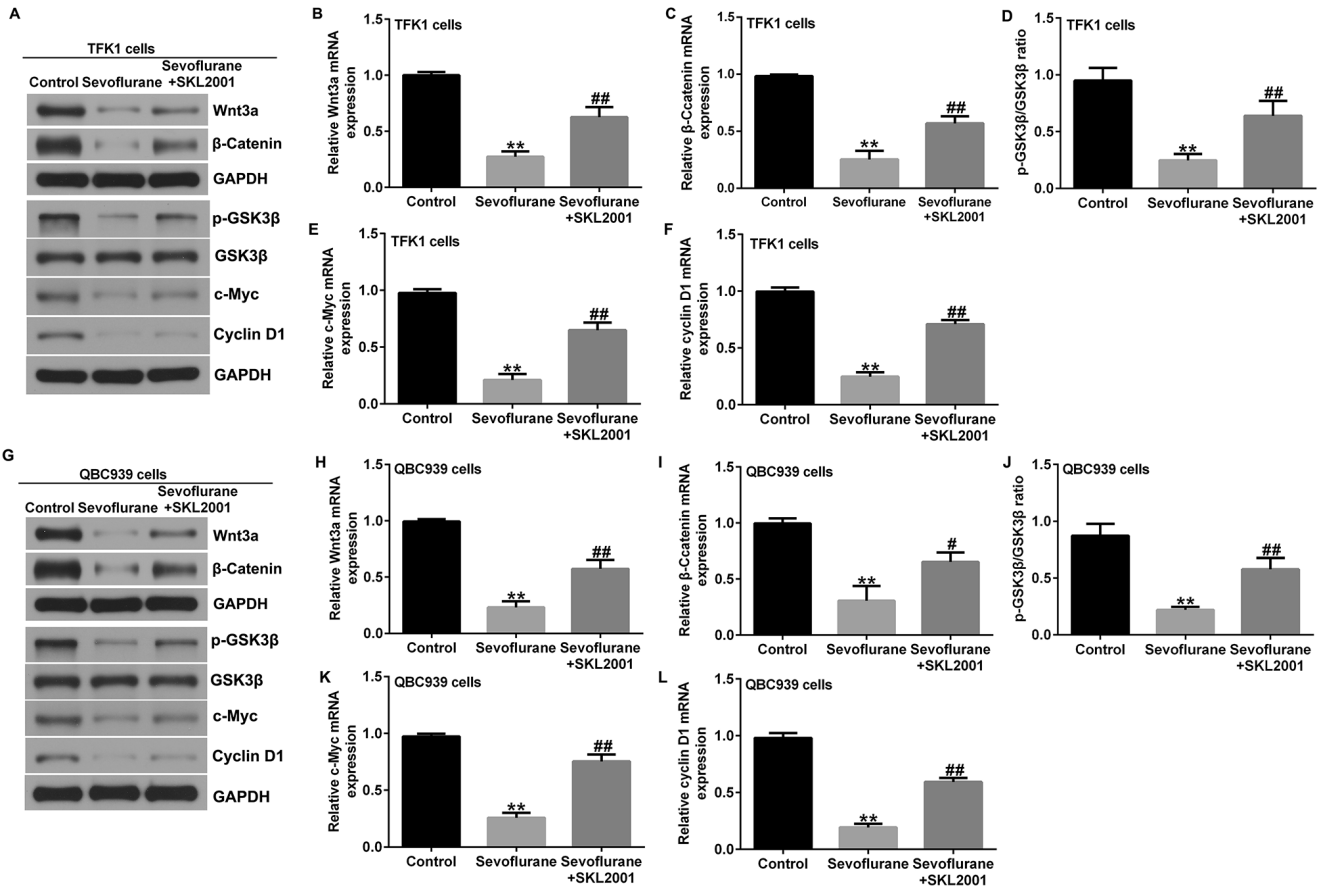
SKL2001 reversed the effects of Sevoflurane on Wnt/β-catenin Signaling. **A-F**. Western blotting and RT-qPCR detected the protein and mRNA expression of Wnt3a, β-catenin, p-GSK3β, GSK3β, c-Myc, and Cyclin D1 in TFK1 cells; **G-L**. Western blotting and RT-qPCR detected the protein and mRNA expression of Wnt3a, β-catenin, p-GSK3β, GSK3β, c-Myc, and Cyclin D1 in QBC939 cells. **p < 0.01 vs. control group; #, ##p < 0.05, 0.01 vs. Sevoflurane treatment group

To further explore the mechanism of action of Sevoflurane in CCA, we treated TFK1 and QBC939 cells with 3.4% sevoflurane for 6 h, followed by culturing with-catenin inducer SKL2001 for 24 h. The inhibition of Wnt3a, β-catenin, c-Myc, and Cyclin D1 protein and mRNA expression, p-GSK3β protein expression, and p-GSK3β/GSK3β ratio in TFK1 cells by sevoflurane was significantly reduced by SKL2001 treatment compared to that in the sevoflurane group (Fig. 5A-F). Similarly, the inhibition of Wnt3a, β-catenin, c-Myc, and Cyclin D1 protein and mRNA expression, p-GSK3β protein expression, and p-GSK3β/GSK3β ratio by sevoflurane was significantly abolished by SKL2001 in QBC939 cells (Fig. 5G-L).

**Fig. 6:**
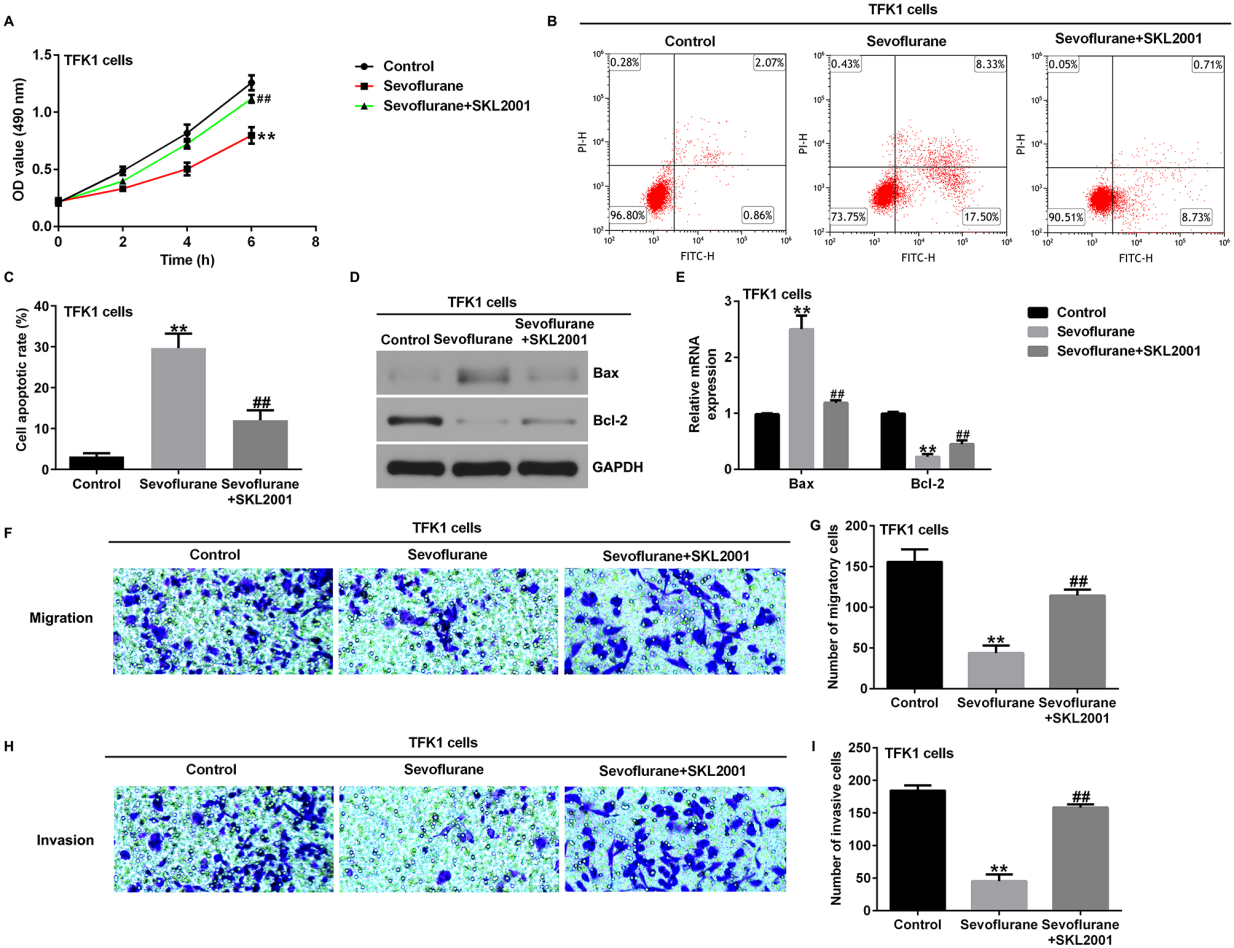
SKL2001 reversed the effects of Sevoflurane on proliferation and apoptosis of TFK1 cells. **A**. CCK-8 assays were used to evaluate the cell proliferation of TFK1 cells; **B-C**. Flow cytometry were used to evaluate the apoptosis of TFK1 cells; **D** and **E**. Analysis of Bax and Bcl2 protein and mRNA expression in TFK1 cells by western blotting and RT-qPCR; F-I. Transwell assays were used to evaluate migration **(F and G)** and invasion (H and I) of TFK1 cells. Magnification: ×200. **p < 0.01 vs. control group; ##p < 0.01 vs. Sevoflurane treatment group

**Fig. 7:**
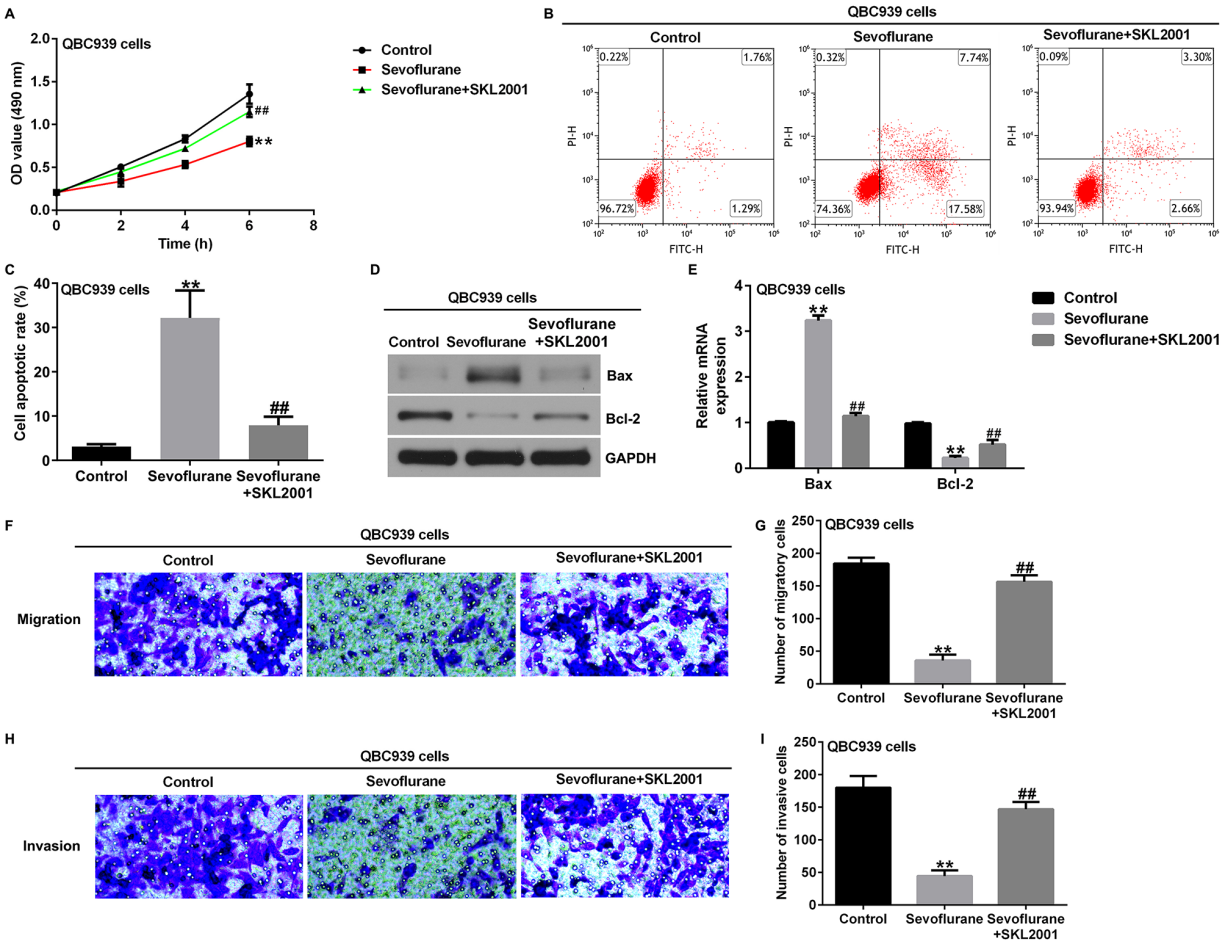
SKL2001 reversed the effects of Sevoflurane on proliferation and apoptosis of QBC939 cells. **A**. CCK-8 assays were used to evaluate the cell proliferation of QBC939 cells; **B-C**. Flow cytometry were used to evaluate the apoptosis of QBC939 cells; **D-F**. Analysis of Bax and Bcl2 protein and mRNA expression in QBC939 cells by western blotting and RT-qPCR; **G-J**. Transwell assays were used to evaluate migration (**F** and **G**) and invasion (**H** and **I**) of QBC939 cells. Magnification: ×200. **p < 0.01 vs. control group; ##p < 0.01 vs. Sevoflurane treatment group

CCK-8 assays showed that SKL2001 abrogated the inhibitory effects of sevoflurane on TFK1 expression and QBC939 cell viability (Figs. 6 and 7 A). In addition, we investigated the effects of SKL2001 on the apoptosis of TFK1 and QBC939 cells. Flow cytometry showed that SKL2001 significantly inhibited apoptosis of TFK1 (Fig. 6B and C) and QBC939 cells (Fig. 7B and C) after treatment with sevoflurane. Western blot analysis and RT-qPCR showed that, compared to the sevoflurane group, SKL2001 co-treatment with sevoflurane significantly decreased the expression levels of Bax and enhanced the protein and mRNA levels of Bcl-2 in TFK1 (Fig. 6D and E) and QBC939 cells (Fig. 7D and E). Transwell assays showed that the inhibitory effects of sevoflurane on cell migration and invasion were significantly eliminated by SKL2001 treatment of TFK1 and QBC939 cells (Figs. 6F-I and 7 F-I). These results suggest the inhibitory effects of Sevoflurane on cholangiocarcinoma cells could be reversed by activating the Wnt/β-catenin signaling pathway.

## Discussion

CCA is a common malignant tumor characterized by high morbidity, high mortality, high metastasis rates, and a poor prognosis [1, 4–6]. The pathogenesis of CCA remains unclear, and an in-depth study of its molecular mechanisms will facilitate the development of new therapeutic approaches. There is growing evidence that sevoflurane plays an important role in tumor development [9, 28]. He et al. revealed that sevoflurane inhibits the proliferation and invasion of colon cancer cells by regulating exosome-mediated circ-HMGCS1 via the miR-34a-5p/SGPP1 axis [12]. In addition, sevoflurane inhibits the proliferation and migration of glioma and intestinal cancer cells and promotes apoptosis [12, 29]. To date, the effects of sevoflurane on CCA have not been reported.

This study is the first to investigate the role and potential molecular mechanisms of sevoflurane in the progression of CCA and to demonstrate that sevoflurane inhibits the proliferation and invasion of cholangiocarcinoma cells by inhibiting catenin signaling pathway. This study demonstrated that the Wnt/β-catenin signaling pathway is involved in the progression of multiple tumors and is a new target for tumor therapy [17]. Previous reports have shown that activation of catenin signaling pathway plays a key role in CCA progression [20]. For example, TTYH3 inhibits apoptosis in CCA through the Wnt/β-catenin signaling pathway [30]. Furthermore, sevoflurane has been found to function in tumors via the Wnt/β-catenin signaling pathway [31, 32]. Recent studies have shown that sevoflurane inhibits the proliferation and invasion of osteosarcoma cells by targeting the miR-203/Wnt/β-Catenin axis [33]. However, the relationship between sevoflurane and the Wnt/β-Catenin signaling pathway in CCA is unclear.

In this study, we found sevoflurane inhibited the proliferation, invasion, and migration of TFK1 and QBC939 cells and promoted apoptosis by treating TFK1 and QBC939 cells with different concentrations of sevoflurane. Moreover, the expression of Wnt/β-catenin signaling pathway-related proteins was significantly reduced in sevoflurane-treated cells, and Wnt/β-catenin inducers could reverse the effect of sevoflurane on CCA. These results imply that sevoflurane may be a new therapeutic target for CCA and may be involved in the regulation of CCA through catenin signaling pathway.

However, this study was mainly conducted at the cellular level, which has certain limitations that need to be studied in depth. Data from in vivo studies of sevoflurane would be beneficial for enhancing the reliability of these results. In future studies, we plan to verify the role and mechanism of action of sevoflurane in CCA in vivo by constructing a CCA mouse model.

## Conclusions

Sevoflurane inhibits the proliferation, migration, and invasion of cholangiocarcinoma cells and induces apoptosis of cholangiocarcinoma cells by inhibiting the Wnt/β-catenin signaling pathway. These results revealed the mechanism of action of sevoflurane in CCA and provided a new strategy for CCA treatment.

## Electronic supplementary material

Below is the link to the electronic supplementary material.

Supplementary Material 1

## Data Availability

The datasets used and/or analyzed during the current study are available from the corresponding author on reasonable request.

## List of abbreviations

CCA: Cholangiocarcinoma
ATCC: American Type Culture Collection
PVDF: Polyvinylidene difluoride
BSA: Bovine serum protein
ANOVA: Analysis of variance
