# Correction: Sevoflurane inhibits cholangiocarcinoma via Wnt/β-catenin signaling pathway

**DOI:** 10.1186/s12876-025-04229-8

**Published:** 2025-09-02

**Authors:** Hui Cheng, Qinfang Li

**Affiliations:** 1 Department of Anesthesiology, People’s Hospital of Dongxihu District, Wuhan, 430040 China; 2 People’s Hospital of Dongxihu District, No. 81 Huanshan Road, Wujiashan, Dongxihu District, Wuhan, 430040 China

**Correction: BMC Gastroenterol 23, 279 (2023)**

**https://doi.org/10.1186/s12876-023-02911-3**

Following publication of the original article it was reported that there were errors in Figs. 2, 6 and 7. The incorrect and correct figures are given below. The figure captions and in-text citations in the original publication are correct.

**Incorrect Fig. 2**.


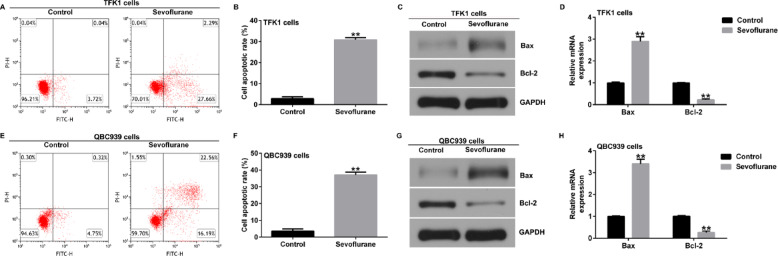


**Correct Fig. 2**.


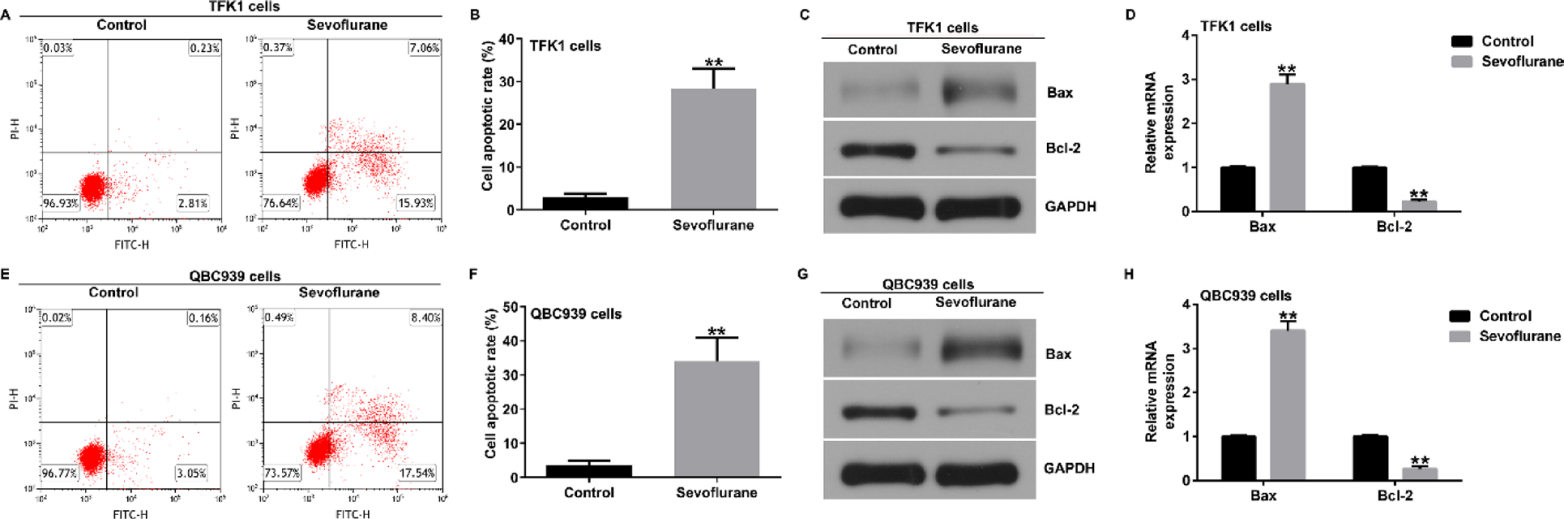


**Incorrect Fig. 6**.


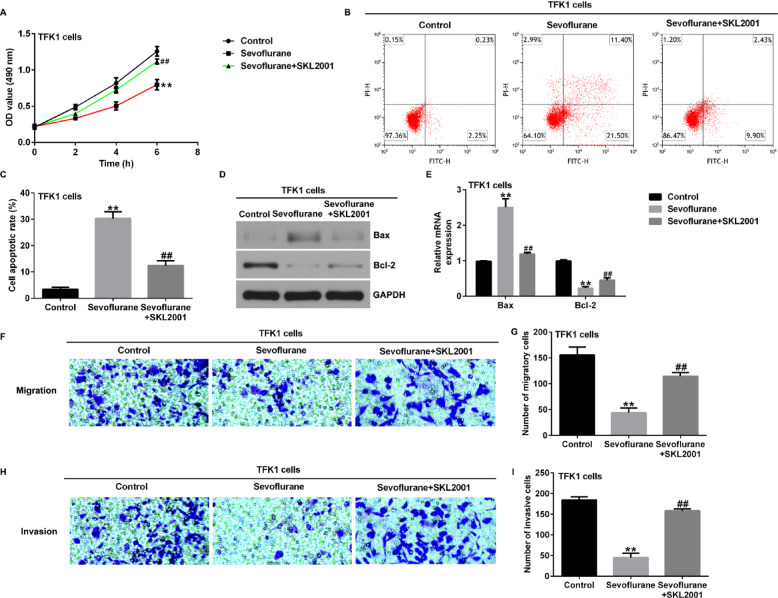


**Correct Fig. 6**.


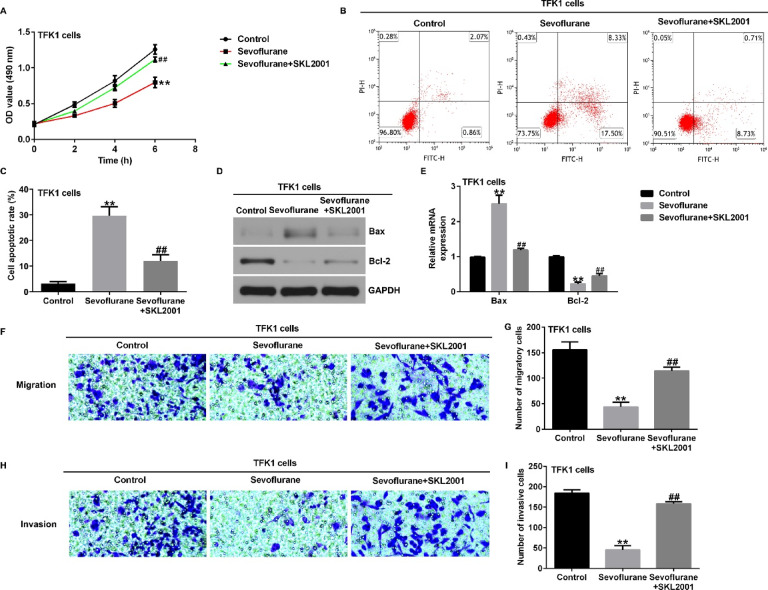


**Incorrect Fig. 7**.


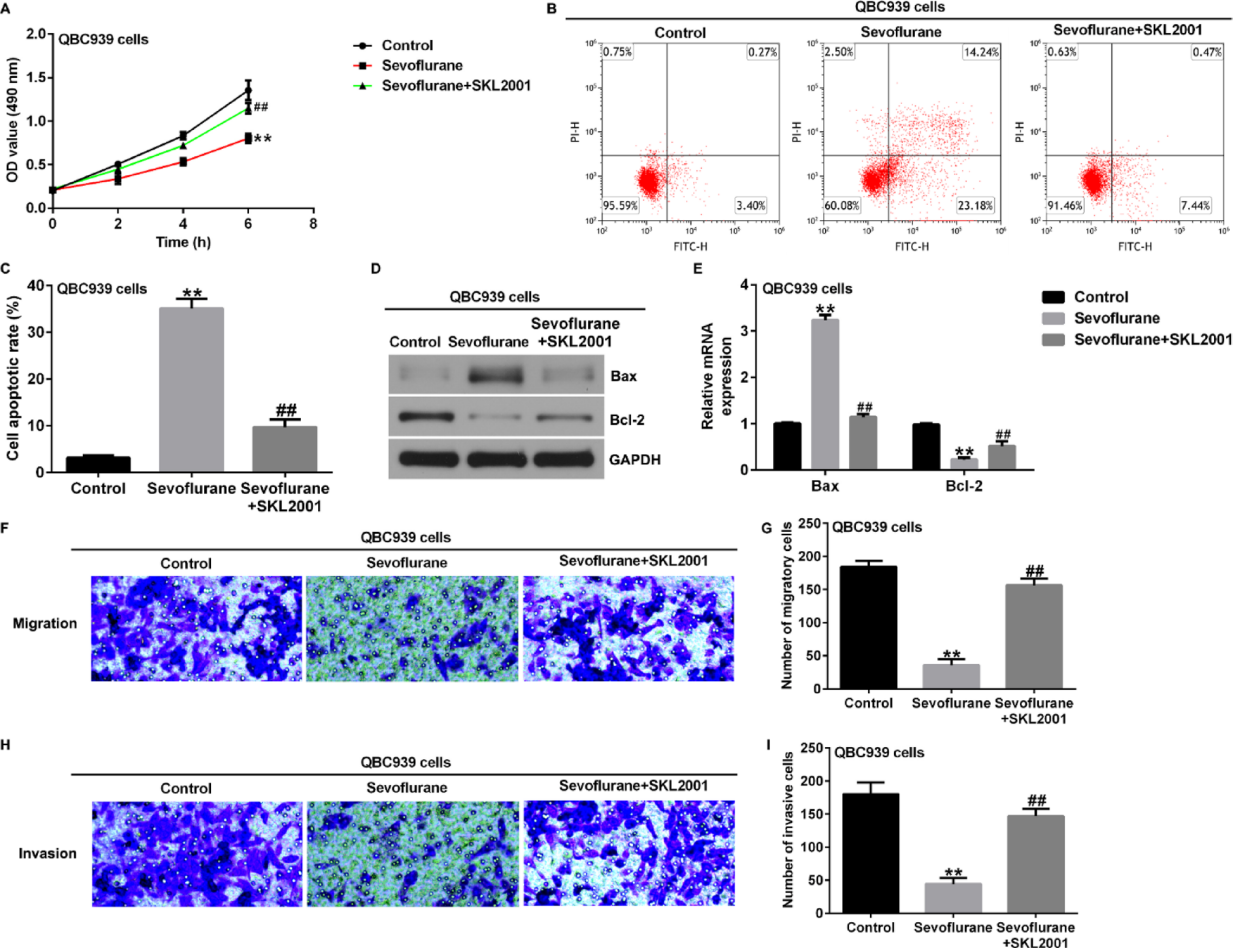


**Correct Fig. 7**.


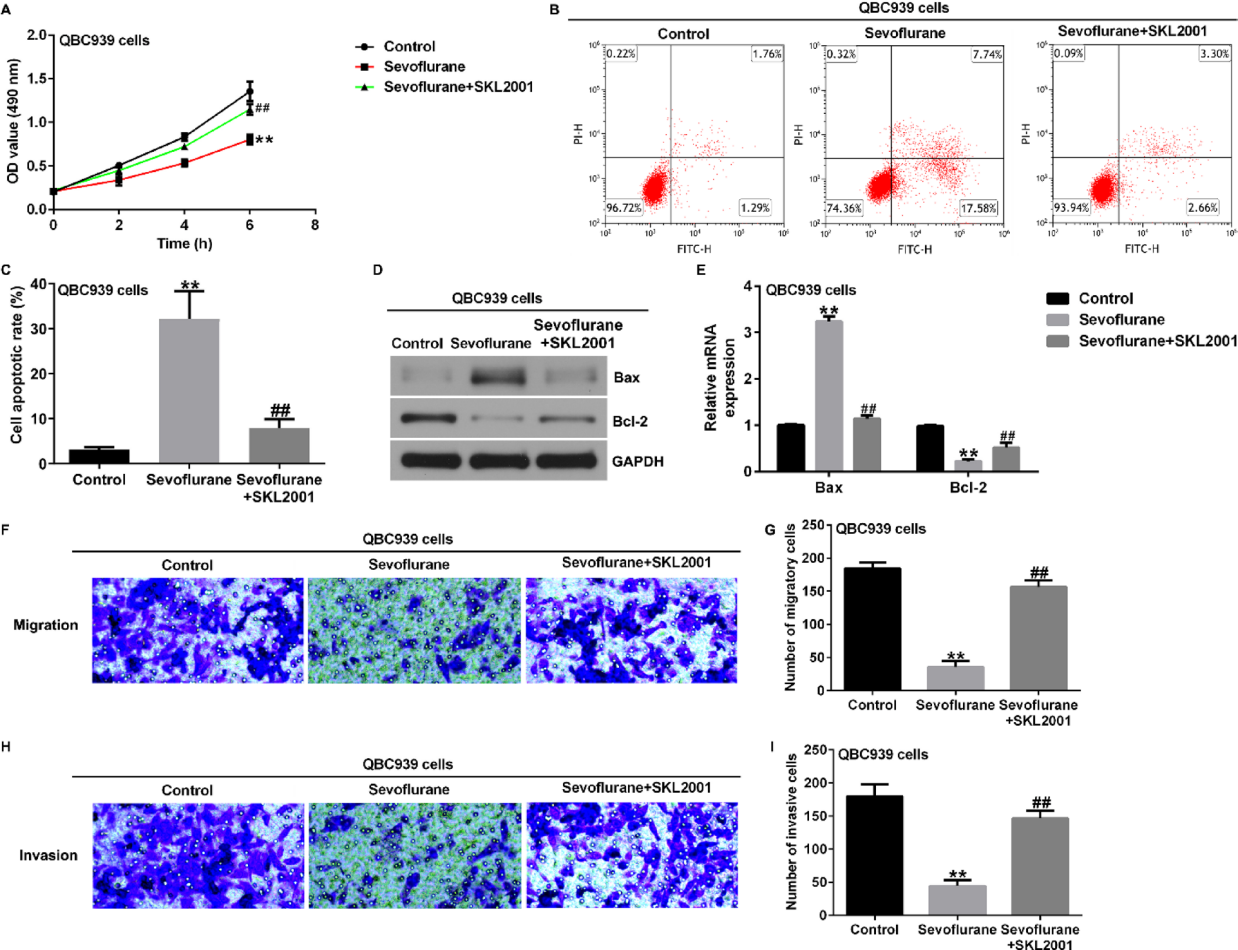


The original article has been updated.

