# Supplementary material for: Sevoflurane inhibits cholangiocarcinoma via Wnt/β-catenin signaling pathway
Source: BMC Gastroenterol. 2023 Aug 11;23:279. doi: 10.1186/s12876-023-02911-3 (PMC10422733; doi:10.1186/s12876-023-02911-3)
Supplement: Supplementary file 1 — Supplementary Material 1 [file 12876_2023_2911_MOESM1_ESM.docx]

**Original blots for Western blot assay**

**Note:**

It should be noted that during the Western blot assay, we first cut out the corresponding membrane according to the molecular weight of the target protein and then incubate with the primary antibody. Therefore our original imprint is not a full film.

**Figure 2C**


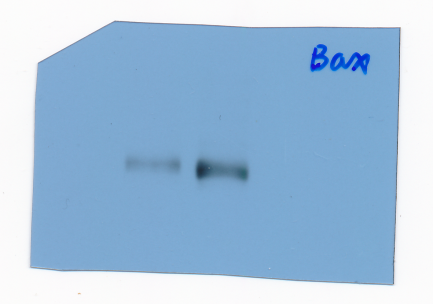


Bax (20 kDa)


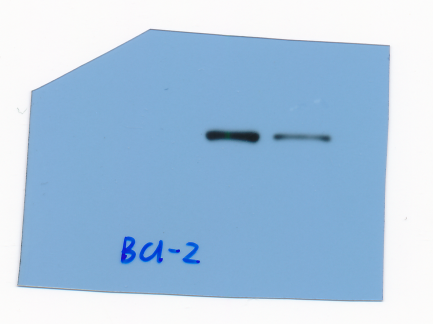


Bcl-2 (26 kDa)


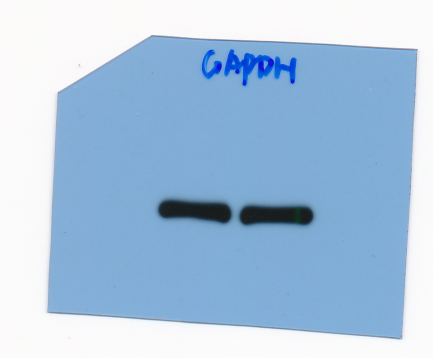


GAPDH (37 kDa)

**Figure 2G**


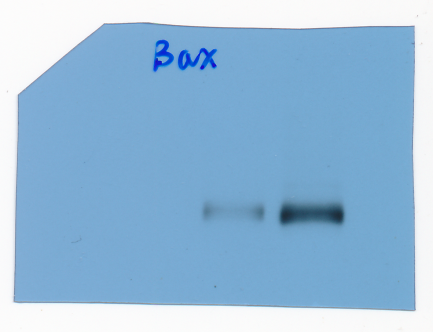


Bax (20 kDa)


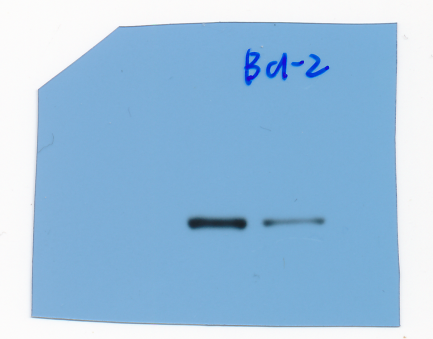


Bcl-2 (26 kDa)


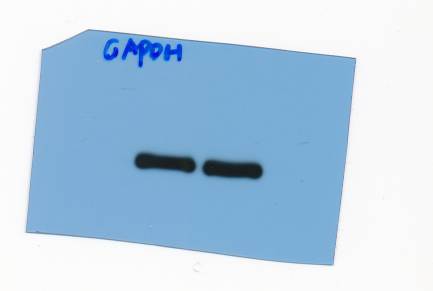


GAPDH (37 kDa)

**Figure 4A**


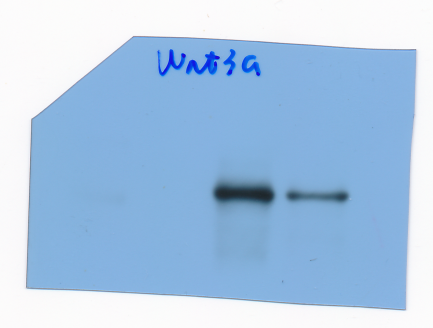


Wnt3a (42 kDa)


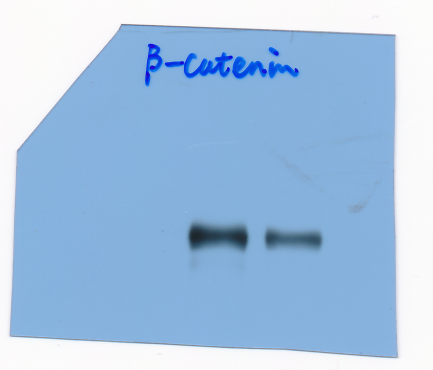


β-Catenin (92 kDa)


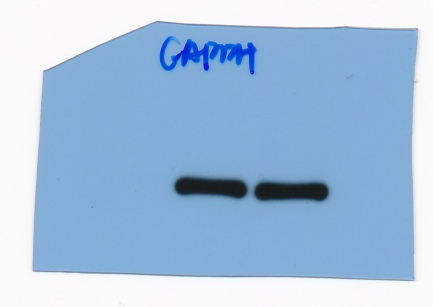


GAPDH (37 kDa)


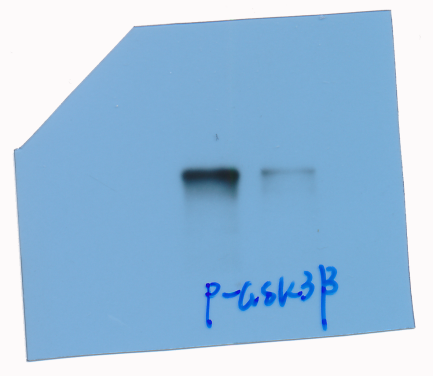


p-GSK3β (46 kDa)


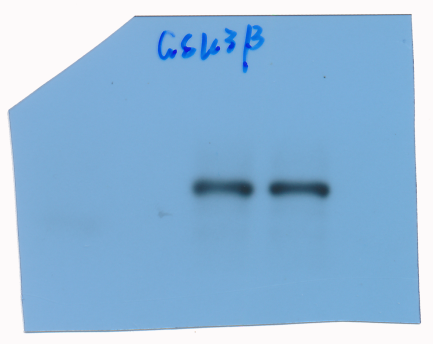


GSK3β (46 kDa)


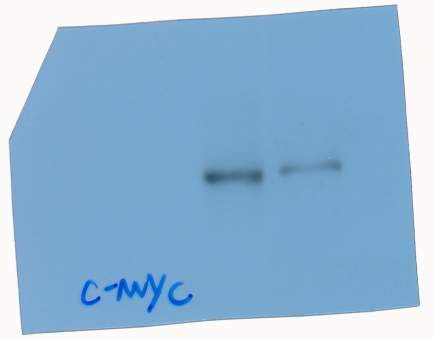


C-Myc (57 kDa)


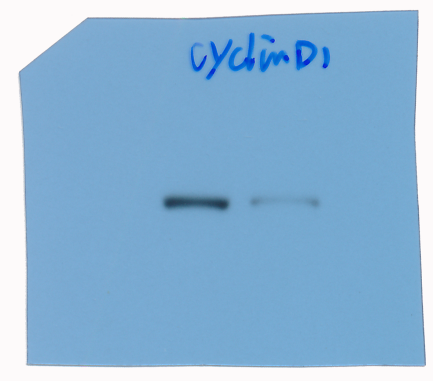


CyclinD1 (34 kDa)


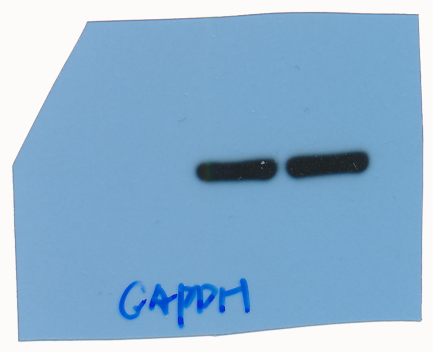


GAPDH (37 kDa)

**Figure 4G**


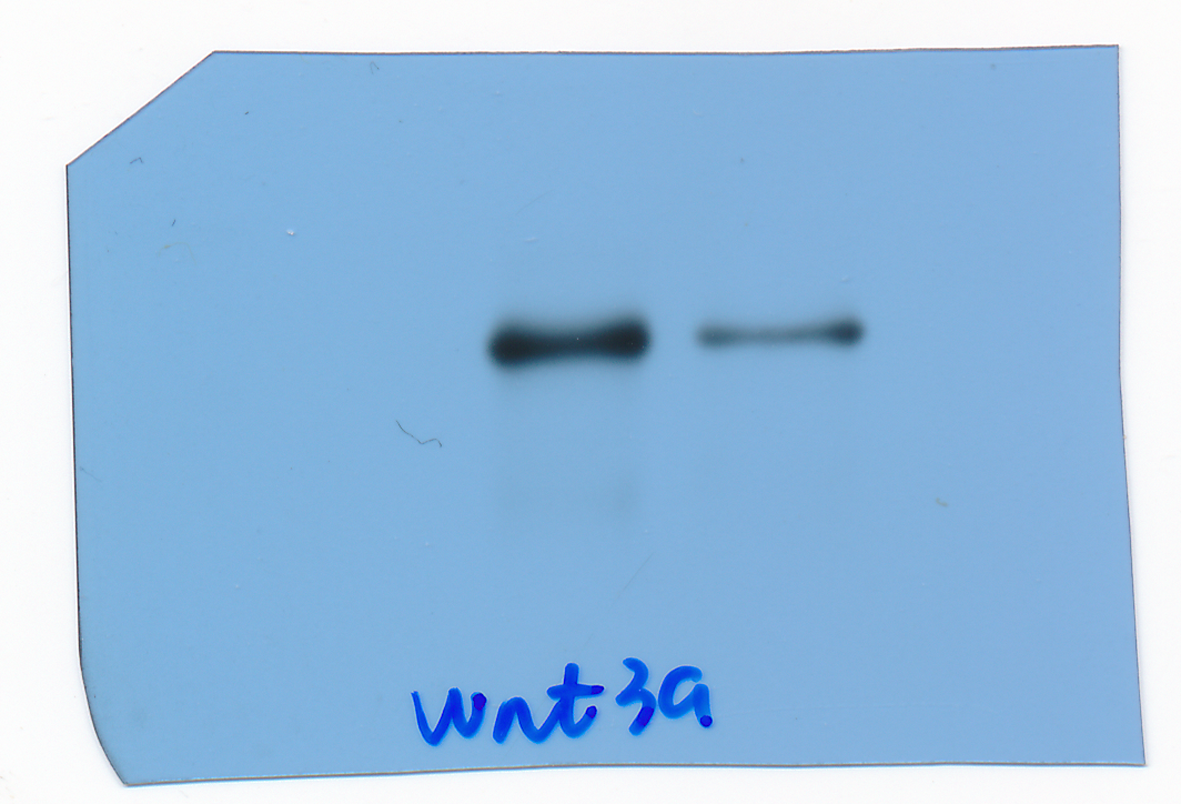


Wnt3a (42 kDa)


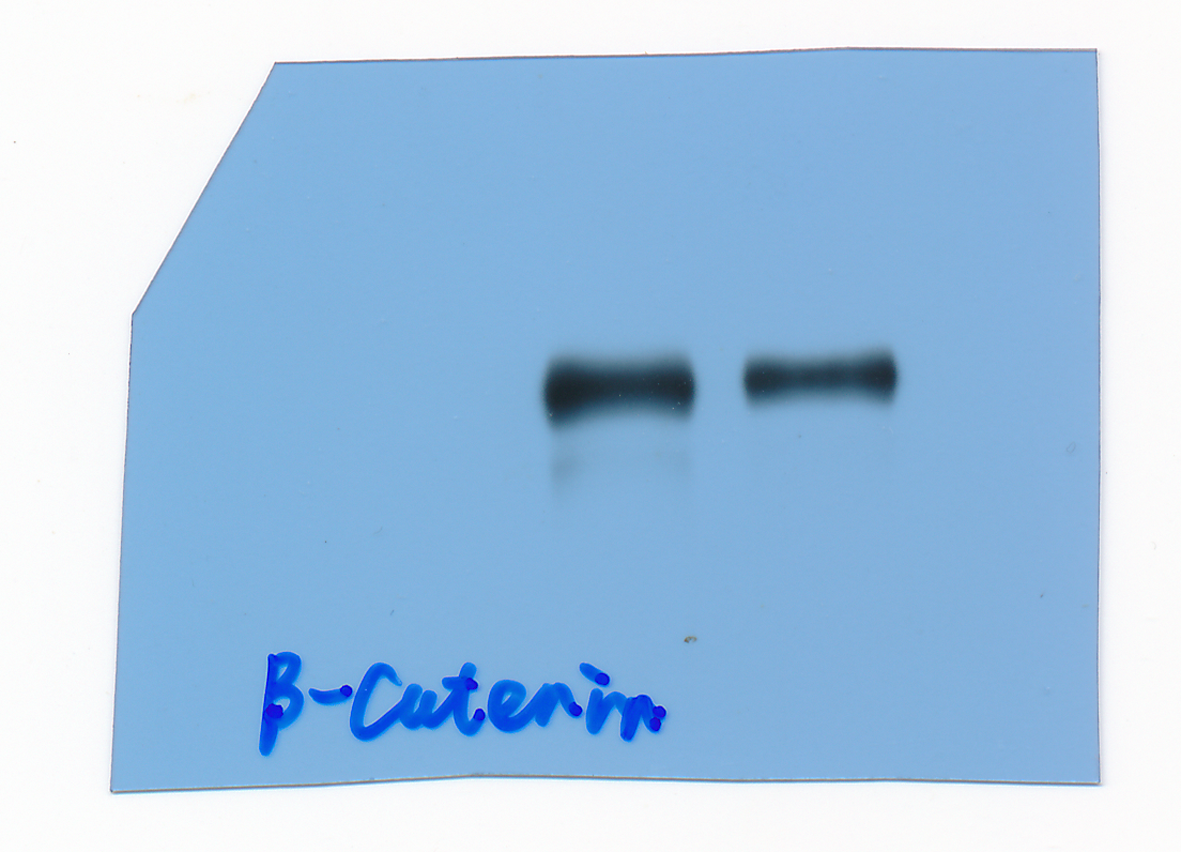


β-catenin (92 kDa)


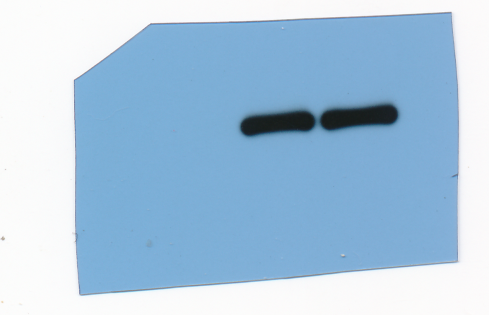


GAPDH (37 kDa)


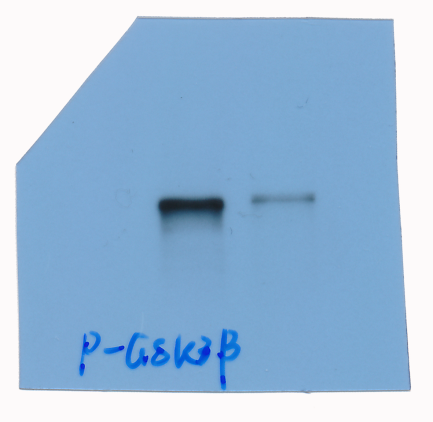


p-GSK3β (46 kDa)


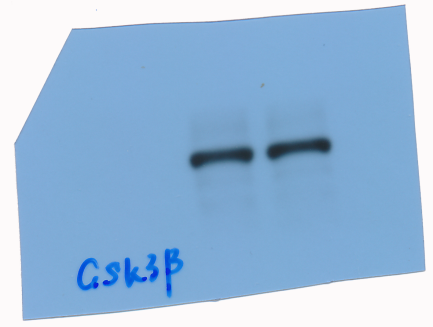


GSK3β (46 kDa)


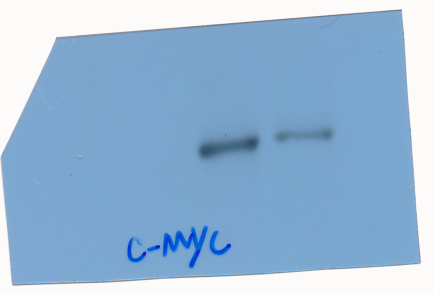


C-Myc (57 kDa)


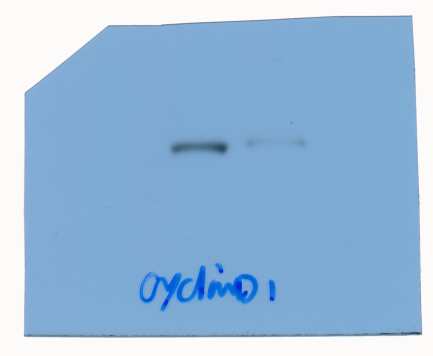


CyclinD1 (34 kDa)


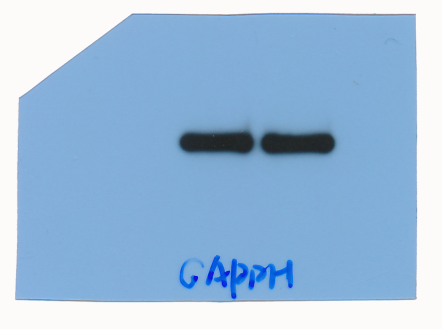


GAPDH (37 kDa)

**Figure 5A**


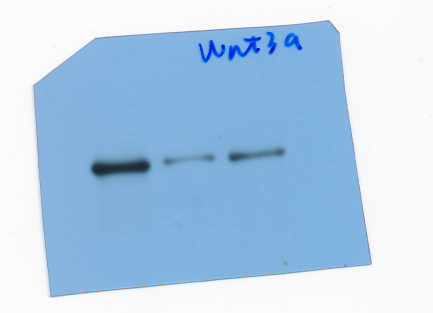


Wnt3a (42 kDa)


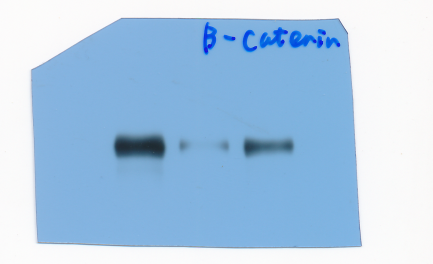


β-catenin (92 kDa)


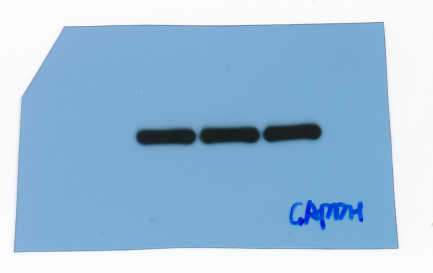


GAPDH (37 kDa)


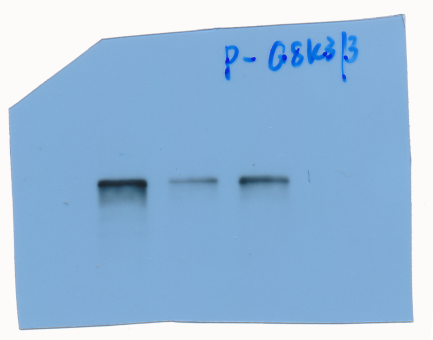


p-GSK3β (46 kDa)


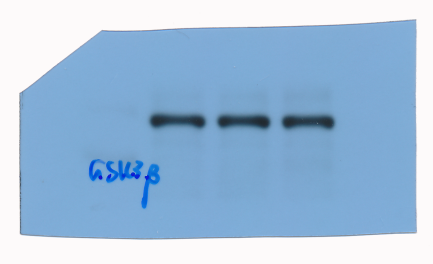


GSK3β (46 kDa)


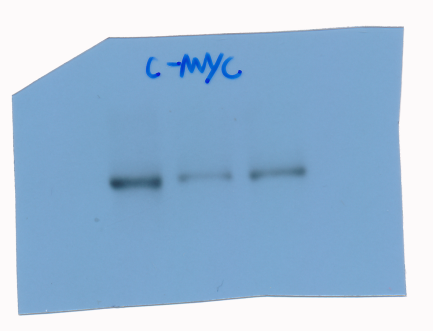


c-Myc (57 kDa)


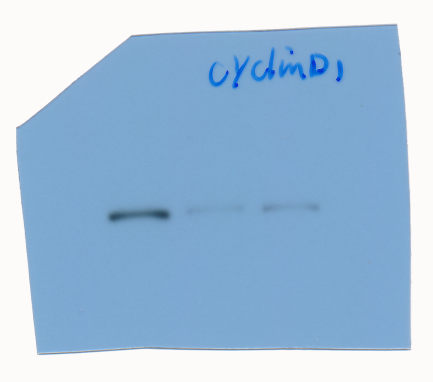


Cyclin D1 (34 kDa)


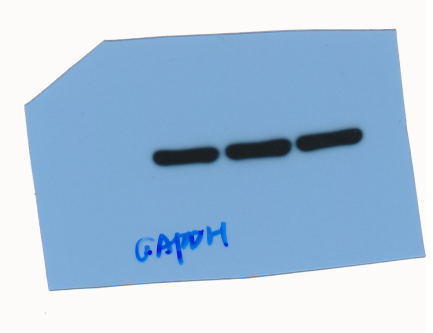


GAPDH (37 kDa)

**Figure 5G**


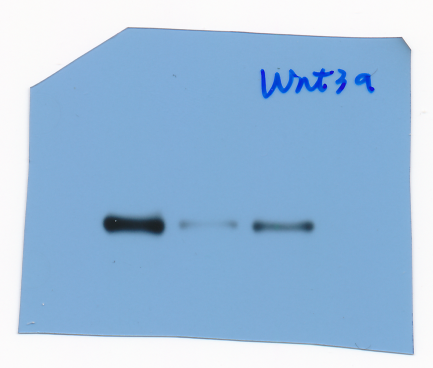


Wnt3a (42 kDa)


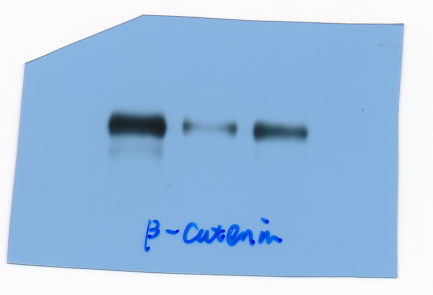


β-catenin (92 kDa)


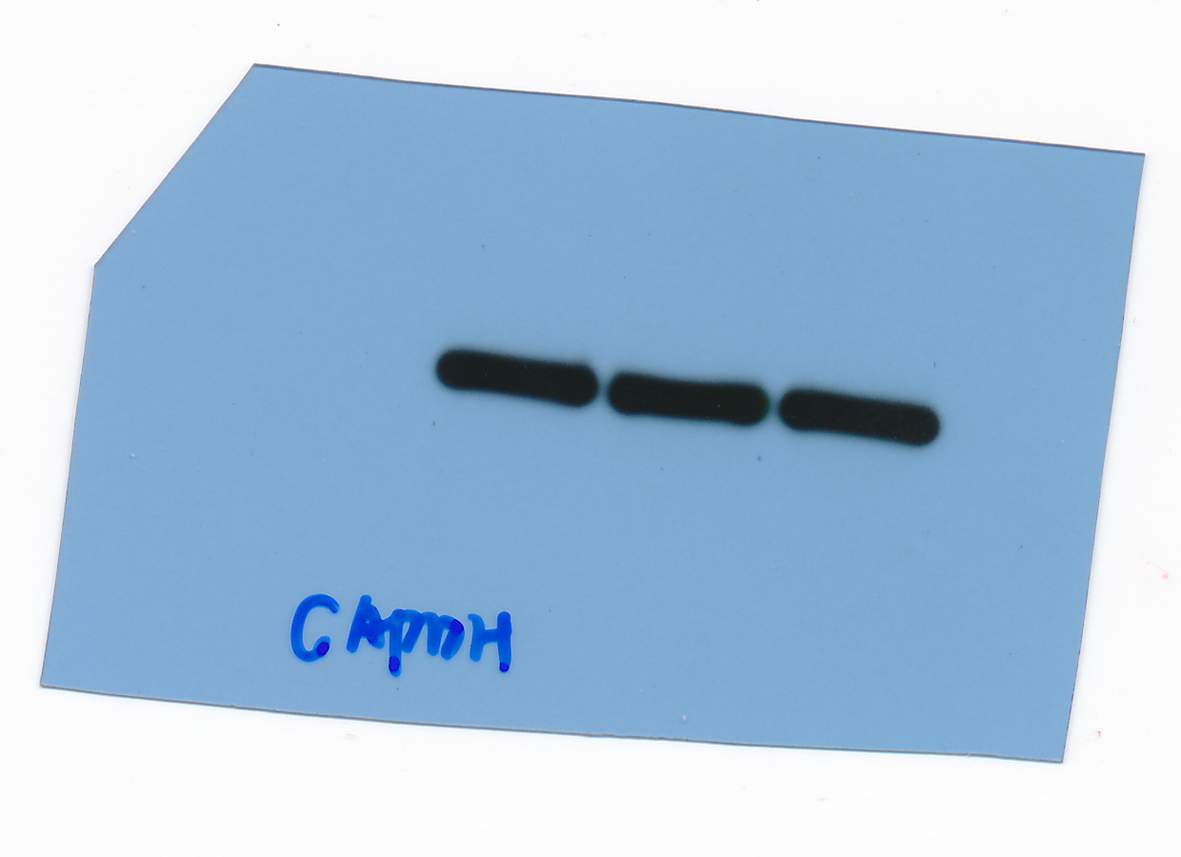


GAPDH (37 kDa)


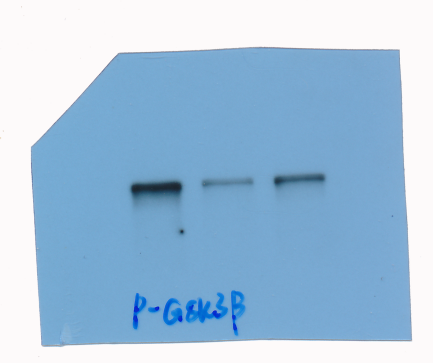


p-GSK3β (46 kDa)


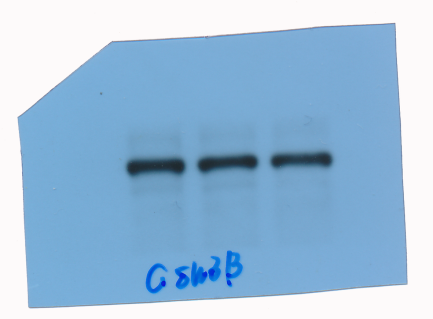


GSK3β (46 kDa)


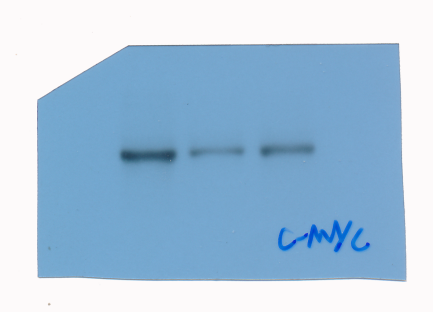


c-Myc (57 kDa)


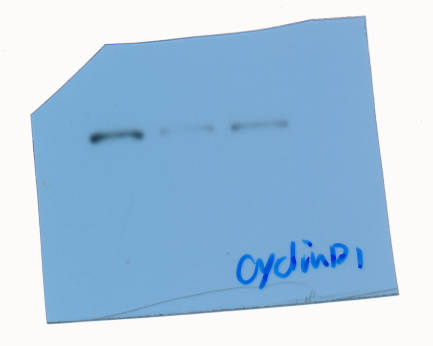


CyclinD1 (34 kDa)


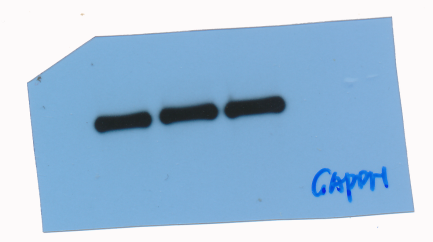


GAPDH (37 kDa)

**Fihure 6**


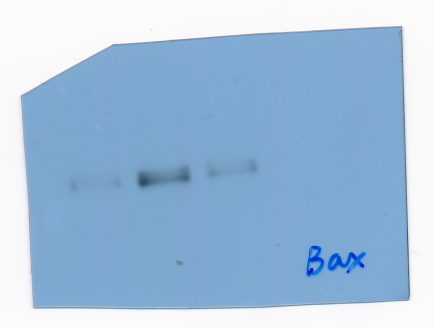


Bax (20 kDa)


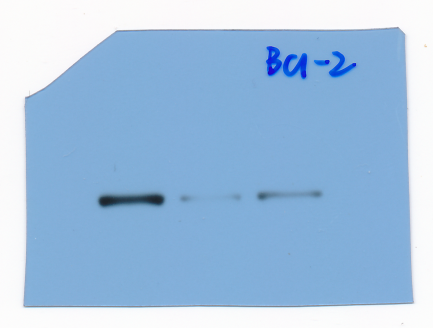


Bcl-2 (26 kDa)


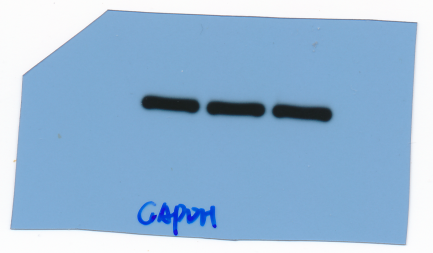


GAPDH (37 kDa)

**Figure 7**


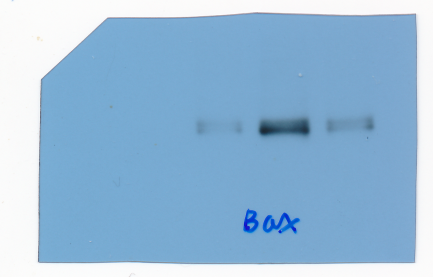


Bax (20 kDa)


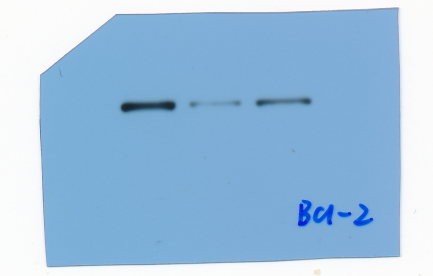


Bcl-2 (26 kDa)


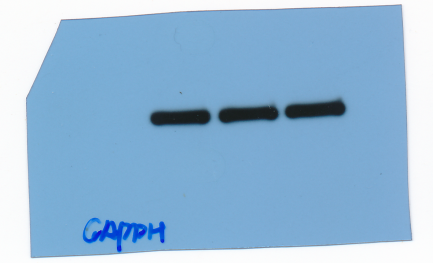


GAPDH (37 kDa)
